# From connectomes to projectomes: an interview with Clay Reid

**DOI:** 10.1117/1.NPh.9.4.040401

**Published:** 2022-11-10

**Authors:** Prakash Kara

**Affiliations:** University of Minnesota, Department of Neuroscience, Minneapolis, Minnesota, United States

## Abstract

Neurophotonics Associate Editor Prakash Kara (Univ. of Minnesota) interviewed his colleague Clay Reid (Allen Institute for Brain Science) about his pioneering work in neuroscience.

**Figure:**
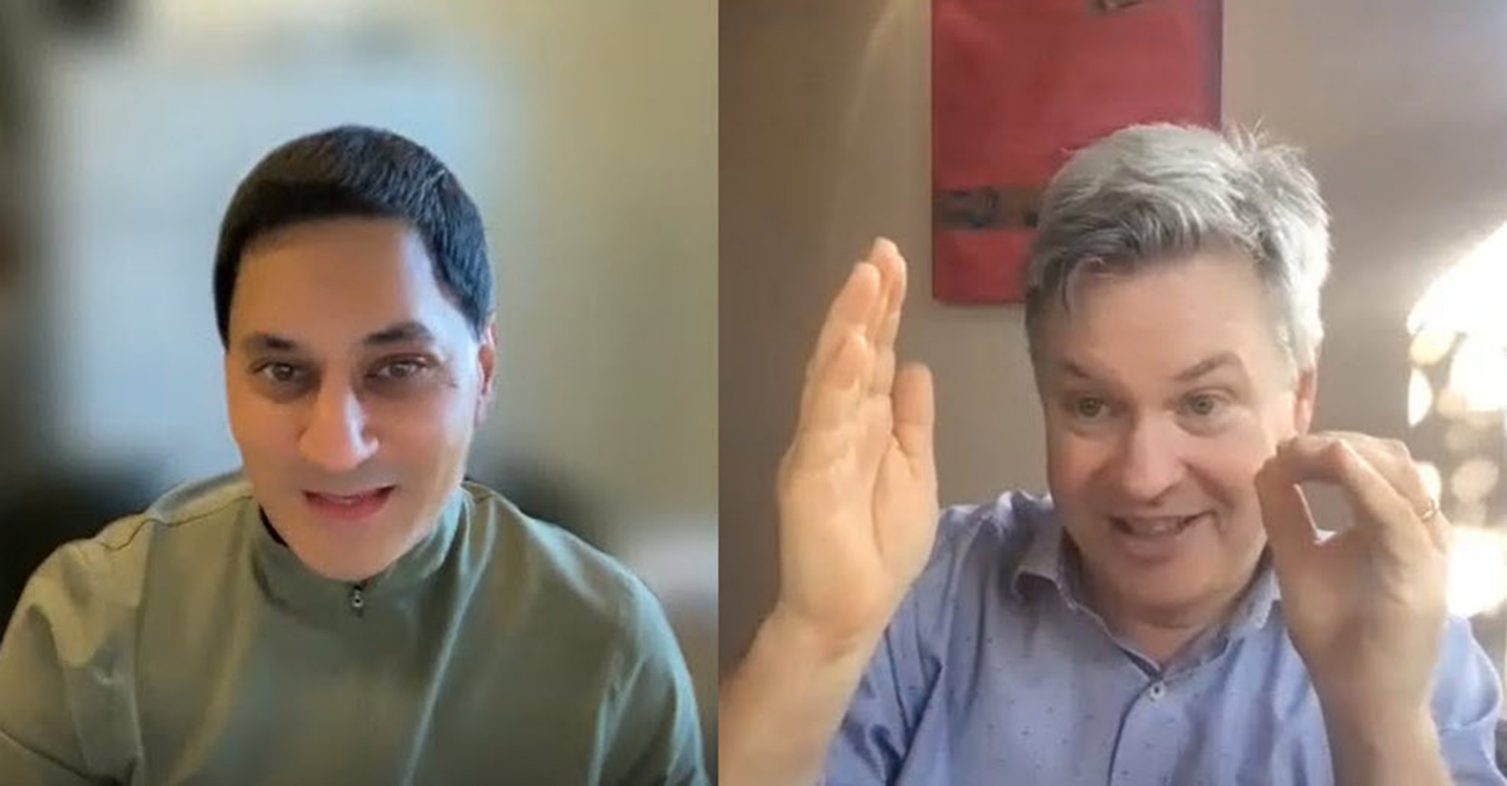
*Neurophotonics* associate editor Dr. Prakash Kara (left) interviewed Dr. Clay Reid, Senior Investigator at the Allen Brain Institute. Readers are also invited to view the interview in video format, https://doi.org/10.1117/1.NPh.9.4.040401.s1.

Prakash Kara: Hello, everyone. My name is Prakash Kara. I’m a professor in the Department of Neuroscience at the University of Minnesota. I’m also one of several associate editors at *Neurophotonics*. One thing I really love about this journal, thanks to our editor Anna Devor, is that we get to interview scientists who are developing the cutting-edge photonic tools and applying them to solve major puzzles in neuroscience. So today I’m very pleased to introduce Dr. Clay Reid, who is here with us. Clay is a senior scientist at the Allen Institute in Seattle. Clay is well funded by the NIH and has broad accomplishments across different topics in neuroscience, which we’ll hear about today.

Among his many accolades, Clay won the Society for Neuroscience Young Investigator Award in 2001 and has published 12 papers in *Science* or *Nature*. Yet Clay has always been very kind and humble – something that I have firsthand experience with because I was lucky enough to be a postdoc in his lab when he was still at Harvard some years ago.

But before we get into the discussion of photonics and other tools – Clay, welcome! Perhaps you can tell us a little bit about how you got interested in neuroscience, because I remember that you have very broad interests, from music – you’ve played cello with Yo-Yo Ma many times – you’re a medical doctor, not practicing, but you also studied philosophy, math, and physics. So how did you get into neuroscience?

Clay Reid: I’m a strictly amateur cellist and I was just lucky from friends to play with Yo-Yo Ma – once, just to correct that. But thank you, Prakash. This is a great opportunity. And thanks for those nice words.

How did I get into neuroscience? Growing up, I wanted to be an academic, a mathematician or physicist. Every once in a while, I thought philosophy might be interesting. But I really thought I was going to be a mathematician or a physicist. I guess I had a decent amount of familial pressure, or sort of the environment of lots of doctors in the family. And I wasn’t sure whether I wanted to do physics. It just seemed like a very tough row to hoe. So, I took a year off after graduating from college. And it’s funny how little things change your life. I think I’ve shared this story several times: to a philosophy professor of mine, I just vaguely said I wanted to perhaps go to medical school, perhaps study the brain, and he said, “oh, Clay, that’s amazing – many of the things you’re interested in – you should really look at the work of Hubel and Wiesel.” And Hubel and Wiesel are, of course, the great pioneers of cortical physiology of the visual system. And I went and read David Hubel’s chapter on vision in *Scientific American* and I said, this is just fantastic! That same summer I read David Marr. This was in the early ‘80s, David Marr’s book on theory behind visual processing – and I just got hooked! I applied to graduate school and found sort of the ideal lab for me. It was just a bunch of ex-mathematicians and physicists, the original Hartline–Ratliff lab at the Rockefeller University where I worked at – wow, a total of probably eight years – with a fantastic advisor, Bob Shapley, both for my graduate work and I did a short postdoc with him even before going back to finish my medical school studies at Cornell. But just a chance conversation with a philosopher sort of set me on my way to studying vision.

Kara: Yeah, that’s amazing. And I think you got to work with Torsten Wiesel directly?

Reid: Exactly. Almost exactly the same time that I got to Rockefeller, Torsten Wiesel moved his lab from Harvard to the Rockefeller University. I didn’t do my PhD in his lab but I ended up doing a postdoc in his lab immediately after I finished medical school. And actually, frankly, that’s where I started doing optical techniques. I did my postdoc with Dan Tso who, following on the work of Amiram Grinvald, was one of the pioneers of optical imaging, of just wide-field microscopy, sort of the surface really using an oxygenation signal, as you very well know, Prakash. I started doing that work but I didn’t publish much during that period. I really at that time was an electrophysiologist. But that’s when I got my start in using optical methods in neuroscience.

Kara: Right, yeah, I remember a little bit of that. But then it seems the biggest jump for you into optical imaging, at least cellular resolution optical imaging, was in 2005 with your paper in *Nature* that has been cited well over 1200 times – probably more by now. So I don’t know if you want to use that as a springboard to tell us about what you are doing in your current lab. And it’s up to you, if you want to tell us what the state of your current lab and research program is, or whether you want to give us a brief tour from that 2005…

Reid: Yeah, let me start a little bit earlier, actually.

Kara: Okay.

Reid: I gather that these interviews are useful both to hear about the course of careers, but also just as advice to, I guess, younger scientists about how to go about building a career. And actually, what really started me in optical techniques was a summer course, the very first year that imaging structure and function in the nervous system was taught at Cold Spring Harbor labs by Rich Lewis at Stanford and the late Larry Katz. And together, they started this fabulous course that just so many people – including you, I think – did you take that course?

Kara: Yes, I did.

Reid: So many people either started doing optical techniques in that course – Karl Deisseroth took it the next year, the second year David Kleinfeld, who did another one of these, certainly taught at that many times. But it was an introduction for me to optical techniques, and we just did it all! So if it’s any advice to young people, if you possibly can, take the summer courses at places like Woods Hole, Cold Spring Harbor. Because they really change your life. They introduce you to many people in whatever field you’re in. But give you just a boot camp in something that can be radically new. And for me, it changed my career a little bit more than a decade later. It just sort of was in the back of my mind for a decade. And what is “it”? It is calcium imaging. I took the course, at the very beginning, very close to the beginning of using calcium imaging to study physiological activity in living brains, or in isolated slices, for instance.

And we – three of us, Rachel Wong at UW now, Beverley Clark at UCL, and I, tried doing calcium imaging of the salamander retina one summer. And who knows if it worked but it really changed the careers of all three of us and again some of the other people. So, I had this idea that I really wanted to do imaging.

And I’m going to roll back a little bit more – apologies if this is too discursive. Really, from the very beginning of my life in neuroscience, I was interested in the relationship between structure and function, between brain connections and physiology. And for the first 15 years of my career, starting in 1983, almost 20 years, until a little bit after 2000, I was an electrophysiologist, as you well know. We did electrophysiology together at the beginning. And we did just every possible variation of recording from neurons at various places in the brain and in the visual system, trying to build upon what we already knew from countless studies – many of them from Hubel and Wiesel, and Shapley’s schools – of what neurons do in the visual system, and using techniques, known as cross-correlation that others had done as well, to find the signature of connectivity between neurons that we knew the physiology.

So it really was structure–function: what does something compute, how does it compute it as evidenced by connections. So those experiments were fun, but they got really frustrating, didn’t they? They got too hard. They go to the next level. And then the question was what next? If we want to study the relationship between physiology and anatomy, wouldn’t it be nice to see the neurons? Wouldn’t it be nice to have an anatomical view from the very beginning of the neurons? And then find, magically, some way of finding the physical connections between the neurons that one looks at, you know, probes their physiology with optical techniques.

So yeah, you were there at the very beginning when we started – we made the transition from pushing 20 years of electrodes in the brain, to optical techniques. And as our discussion was before we started recording, how do you balance doing things safely and swinging for the fences. And at the end of the electrophysiology period, we knew how to do this. We could get papers and just continue turning the crank. But when you get tenured, you say, “now or never! They’re not going to fire me. I have some grants that are a little bit topped up. I can – I’m not going to starve, or the people in my lab are not going to starve for the next three, or four, or five years. Let’s do something crazy!” If your friends are people at Bell Labs like Winfried Denk and Rafa Yuste and Karel Svoboda, the thing you want to do is calcium imaging.

So we bought, with NIH money, a two-photon microscope. We hooked it up. We turned it on and started looking at the brain of rodents. And started off really not knowing what we were going to do exactly, hoping to do some calcium imaging, but just to get started we were just looking at GFP, YFP in the brain. And, you know, luck favors the prepared. We were prepared to record from the brain. And then Arthur Konnerth, just as soon as we were set up, published that fabulous paper that allowed us to – first him, and then us, and then others – to fill up using a chemical indicator, Oregon Green BAPTA-1-AM, get calcium indicator in cortical neurons, and record from the living brain. And that was one of the most – that was probably the most fun time I’ve had in the lab. It was just crazy. I mean, you remember it, right?

Kara: Yeah, of course. yeah.

Reid: It was all these years learning how to tease out the secrets of what a cortical neuron in visual cortex does. I sound hopelessly old fashioned when I say this but cortical neurons – visual cortical neurons – respond to vertical, or horizontal, or oblique bars. And we’d always listened to those in a dark room, sort of imagining the black box. And then all of a sudden, we could just look at the brain. And put a bar in front of the animal’s eyes, and watch the brain light up! All the neurons that responded well to a vertical bar went pop! And I was excited.

Kara: I think the precision of the direction fractures, it’s just something out of this world. I think we couldn’t believe that you could draw a straight line that separated one cell body over one direction and the cell body across that was the opposite direction.

Reid: It really was amazing, wasn’t it? We made movies, or the brain and the microscope made the movies, of what the cells did. And we could have made a caricature of what we would see, beforehand. But our caricature would not include a sea of neurons that wanted to see bars moving this way, another sea of neurons wanting to see that way – and exactly – just, you move 20 microns one way or another on a previously invisible boundary. And to this day, we don’t really understand that. To this day, we do not understand that. The axons and dendrites of those cells are much larger than that imaginary boundary.

Kara: That was quite a fascinating period, clearly. But then something else happened. You had other crazy ideas.

Reid: Yeah, it’s funny. Tenure, the NIH, and big – it’s always good to do the right thing and make a major shout out to the National Eye Institute – if we start talking about “big science” I’m going to be showing an inconsistency. But back then, the National Eye Institute – and to this day, the National Eye Institute funds – the main thing it does is fund individual researchers RO1s and keeps the field alive so people are able to capitalize on serendipity, or just straightforward science. And the NIH funding continued, allowing us to go to the next stage and be a little bit crazy.

So I said, why would – why scientifically, not just wanting to see beautiful movies of the brain thinking – but why scientifically, did it make sense given, what the lab did, to do optical recording? It was allowing physiology to become anatomy. But the big question was, what’s the connectivity, what’s connected to what. And electrophysiology can do that poorly, with timing. Calcium imaging is not going to give you that. But, once you’re seeing neurons, you can do anatomy. And what were the thoughts back then? What are the thoughts today? A big thought was doing viral tracing, trend synaptic viral tracing. And that was one of the plans. But we kept our ears to the ground. And again, Winfried Denk, who gave us the tools of two-photon microscopy – together with David Tank, and Rafa, and Karel Svoboda, and others – combined it with calcium imaging. Winfried, at beginning of the 2000s, started working on the old mature field of serial section electron microscopy. And that’s a field that had been around since the mid ‘50s, of taking sections of brain, imaging them with cameras, taking 100 sequential sections, or 1000 sequential sections, or even more, building up books of these large electron micrographs, and reconstructing three-dimensional models of the neuropil.

So, people had done this famously in the worm, famously in the retina, famously Murray Sherman in the thalamus, and others. But Winfried Denk said, let’s bring this to an industrial level. Let’s not – it had already been computerized, and people like Kristen Harris had been using computers for doing three-dimensional reconstructions – but Winfried said, let’s do this at a very large scale. And he built a wonderful system with electron microscopy: take a brain, fix it really hard, impregnate it with heavy metals that go to the lipid bilayers and proteins, the cell membranes, importantly. Typically, you’d cut a section, painstakingly put that section on a bed of ultrathin plastic, and image it through a transmission EM. Winfried did the other approach of “let’s cut a section, throw it away, look at the cut surface” – and that has a lot of – the registration problem is easier, etc., etc. – but Winfried computerized this, automated it, and by 2004 had these fabulous movies of serial sections over larger volumes than people had looked before.

At that time, a number of us, a small number of us, got the bug. The small number of us were Robert Mark, in the retina, Winfried, of course, Jeff Lichtman at Harvard College, and our group. And perhaps, one or two others, at the very beginning. And I was lucky enough to have a fabulous graduate student, Davi Bock at Harvard. We all know, you know. And together, we just went kind of nutty and decided let’s do electron microscopy too. Let’s build a crazy camera system and –

Kara: The rest of us in the lab did think the two of you were crazy.

Reid: We were demonstrably crazy! You were quite accurate. We had a lot of things going for us. You know, Davi is a monster, experimentalist monster, computer person – and Elio Raviola, the best neighbor, scientific neighbor, anyone could ever ask for, was my neighbor at Harvard. And he taught me but he really taught Davi how to be a first-rate electron microscopist. And we just did the thing. Very luckily this wasn’t NIH money. This was money that Jeff Lichtman had raised at Harvard College. And Jeff very generously allowed us to share in this endeavor, the Harvard Connectome Project.

Kara: One quick thing was that part of the crazy was not just the EM, but finding the *in-vivo* imaged cells in the EM. So you very kindly give credit to all these people, but nobody had done that before.

Reid: Yeah, I guess no one had done that before and you know everyone finds their niche. I’ve always loved doing impossible experiments. The electrophysiology got boring by the end but it was hard work. And only with people like you, and originally Jose Manuel, and Marty Usrey, and a few others, only just with incredible hard work could we do that. Same went with the calcium imaging. People were doing it. But it was really hard work to get it to go. And especially for novices, like me, and Kenichi, and you, and Yeang Ch’ng, and Sooyoung Chung. We were all novices. And we just made it up. And yes, then two for two, let’s try for something crazier: do electron microscopy that we hadn’t done but with a lot of help.

But the point of it was not to do electron microscopy, just electron microscopy, it was to relate structure and function. What do neurons do? And how does that relate to how they’re connected? So the only way to do that would be to record from the neurons. And electrophysiology just would never work for this. You had to see them. You have to map them incredibly carefully in the brain. You have to label the blood vessels *in vivo*, compare it to the neurons you’re recording in real time. Keep those vessels labels. Keep the same little chunk of brain. Give it to someone who is just a miraculous experimentalist, Davi Bock or later Wei-Chung Lee, and just do the correspondence problem, because there’s no point in being off by two neurons. If you’re saying this neuron responds to vertical in that direction and it’s connected to another neuron over here that responds to vertical in that direction, if you’re wrong about which neuron it is in the electron microscopy, it’s gobbledygook.

Thankfully the brain is like a fingerprint. Once you get in the neighborhood, you can really see which cell is which. There are some ambiguities, but you can factor those out and say, it’s too ambiguous, I won’t count it. But by and large, you can do multimodal correspondence. And we weren’t – no one had done it before – but we did it simultaneously with Winfried Denk doing exactly the same experiment in the retina. So it was one of these – no one was hiding what they were doing. It was a nascent field. Everyone was talking. Everyone was getting information from each other. We had these early summits where everyone in the field was there. And we’d share with Winfried’s group. He’d share with us. And thankfully we all published together – sort of a happy ending!

Kara: What was one – or two – of the most surprising results from that kind of approach, that you had no idea existed before?

Reid: I wish I could say a surprising result. Winfried found – I’m not an expert in retinal direction selectivity, although I’ve been following it for my entire career. But Winfried found a very nice pattern that related connectivity to direction selectivity. We found – in that first study with Davi, we found something that was not particularly surprising, that a certain – now we know – back then we thought, boy, this is getting too detailed – but it was a story about excitatory neurons and inhibitory neurons. And the inhibitory neurons that we were studying at the time by and large were nonselective for things like orientation, or very weakly selective. And the excitatory neurons were very selective. So the sort of simple – it’s really a null – hypothesis is that there’s no precise wiring with respect to the orientation, or the specificity of a specific class onto a nonspecific class. And that’s what we found. So it was largely a techniques paper. The second paper, we actually did discover a few things. We discovered the very large number of synapses from the beginning of the axons of excitatory neurons onto inhibitory cells.

There really was no hint of that in the literature. And that’s the first thing we found. And really, what it was was a geometrical cell type-specific wiring motif. And thank goodness we reported on it, and made a thing of it and were very careful about it. Because that’s the unexpected discovery. It’s not a great discovery but it’s an interesting thing that we found.

The second paper with Wei-Chung Li, we found a positive result that was also being found with other techniques, but a positive result, about neurons that do the same thing in the cortex are connected to other neurons to do the same thing. Plus – so – well, go ahead, please.

Kara: In the interest of time, maybe you can take us to where things are currently at the Allen…

Reid: Yes.

Kara: And I think the *Neurophotonics* journal audience would be curious to know is it just scaling up, getting through more brains faster, or are there new kinds of questions you’re now asking with industrializing these techniques to even greater extent at the Allen, so maybe you can tell us –

Reid: It’s a great question. I’ll try to be less discursive of it, I think. Again, I guess luck favors the prepared. When these papers started coming out of connecting structure and function, Jacob Vogelstein was a program officer. And then very soon afterwards David Markowitz was the program officer at IARPA – like DARPA, but Intelligence Advanced Research Programs (I think it’s activity, not administration) – put together a program to do precisely that, to scale up that experiment, the structure–function experiment, with the idea – a very classical idea that I will not allow myself to get into because there’s too much to talk about – that given how much cortical anatomy and physiology inspired the first wave of machine learning, in particular, convolutional networks and everything related to them – the idea held by a lot of people that more details and complete knowledge of how cortical networks compute could really help, not wire-by-wire, but help inspire approaches for AI. Let’s leave that aside. But that created a program that allowed us in collaboration with Sebastian Seung and his group at Princeton and Andreas Tolias and his group that Baylor College of Medicine to take the experiment we did, first published in 2011, started in 2006, and scale it up – I don’t know – hundredfold, to do an entire cubic millimeter of cortex, which always was sort of the holy grail of the field. That’s a hyper column of cortex, a complete module of cortical circuitry.

And we scaled it up with the goal of doing really complete structure–function analysis. But for my money, I think, by far the more interesting results are actually reminiscent of the more important results from our first paper. It’s cell-type-specific connectivity. First, when you have the morphology of 70,000 neurons, their dendrites just in detail you can’t imagine, much – not all, certainly – but a decent amount of the axons reconstructed, you can do a morphological classification of cells. So just basic neurobiology. Better than you could imagine. Unbiased, complete, and 70,000 reconstructions at that level is just unheard of.

And then looking at the connectivity of that zoo of different cell types. That’s what the Allen Institute does, especially the current incarnation of the Allen Institute for Brain Science. It’s the neurobiology of cell types. And as a physiologist who wanted to find the Holy Grail of “how does connectivity underlie specific computations,” it’s not a bad day job to unlock these really precise rules of connectivity between different cell types. It’s back to Cajal but with rule upon rule upon rule of how cells are connected to each other.

So that’s an exciting outcome of just going big and saying, Okay, let’s look at that!

Kara: So what about some other offshoots? I mean, for example, you published a paper, *in silico* – the network – *in silico* network of layer 4. Did this come out of this project?

Reid: Absolutely. So I’m talking about our contribution where really our contribution could not exist without initially the segmentation of the data that we collected by Sebastian Seung, the analysis by us and Sebastian’s group, and Andreas Tolias’s group. The paper you’re talking about is really a physiology paper. So while we are concentrating on cell types among other things, Andreas Tolias is concentrating on the physiology they did and its relationship to anatomy. Some of the earliest publications from the entire big project are on the physiology alone. And so the paper you’re talking about is really a paper from Andreas Tolias. We’re all on it. We all discuss it. But it’s fabulous sort of pure computational neuroscience, computational physiology, that they do.

Kara: You mentioned “big science,” and this DARPA grant is clearly mega big science. But all the people you mentioned are established investigators. I mean, is there any hope that a new PI could get in this game?

Reid: It’s a good question. It’s *the* question because I said, early on, we’re going to get to a moment of inconsistency. I wouldn’t have a successful career if it weren’t for the steady funding as a junior faculty member to do modest laboratory science. Now, just by the nature of my research and where I am at the Allen Institute for Brain Science, we do large projects.

Two questions. One: what’s a good portfolio for funding agencies? I think it shouldn’t be one extreme or the other. Some people really would want the big projects to be very few and to have it mostly be laboratory research. I think virtually nobody says it should be all big science. That would be crazy. That would be eating the seed corn. That would be – and it just it’s most discovery. True discovery today obviously comes not from the big projects. Well, who knows – I’ll retract that statement. But most of the big discoveries do come from individuals.

I won’t weigh in myself, as a beneficiary of big science I feel uncomfortable. Certainly, I don’t think there should be more big science. And I do think the pendulum will go back and forth. But there needs to be support for small groups. And there does need to be the possibility of smaller groups working in big science.

Kara: That was my question. A small lab could do – could do their own small RO1 project. But is it going to be easy for them to team up with the big science groups?

Reid: I mean, certainly not everyone is going to benefit from that. So it’s not equal for everyone. Then you could ask the question is it – I don’t know – if the word is “democratic” or “fair,” or something. Can younger scientists contribute? At least to first approximation, yes. I was just yesterday at the kickoff for perhaps the biggest projects that are coming out of the BRAIN Initiative right now, the BICAN awards, the cell census networks of the continuation of the BICCN awards. At this kickoff, there were 200 people. And of course, there were the big labs. There were the big labs, the Broad, Allen Institute, a few others. But there are junior people, untenured people who find themselves in the right place. Of course, they have to be very successful, junior people, finding themselves in the right place where they have the right techniques, the techniques scale, and they have a track record. And they do join these big consortiums. I don’t know if that’s the best argument for it because it’s still the 1%. And it makes you uncomfortable. But in physics, it’s even – or certainly astronomy, it’s more right- or left-shifted. So for biology to find its place, that’s a time for the future. But people have to talk about it.

Kara: Just one quick follow up question on big science: were you equipped to manage big science? It seems unfathomable from an individual lab level how you manage some of these projects, 100 author papers. Does that come naturally, or?

Reid: I mean, for those who know me including you, let’s stipulate, it does not come naturally to me. But let’s look, so far the one – I was going to say “arguably” big science but no, it is big science. This IARPA MICrONS program is big science. And the only way to do it is to have central control. This was largely David Markowitz who knit together groups at three plus sites. And each site needs very strong program project management. That’s one thing that the Allen Institute does fabulously. They have groups, they have a department for program management. And I still haven’t said the names of – there are hundred names to name, but Nuno da Costa really was the driver of our end. I was the uncle, but Nuno was the driver of the project and with Forrest Collman. But Shelby Suckow, who was our program manager – project manager – we would not have been able to do it without tremendous management. But I almost started answering the question by saying it wasn’t that big. At the end of the day there are – at least for this project, the data collection for the IARPA – there’s a core group of seven, eight, nine people who did the difficult laboratory science. And that’s a manageable small group doing something hard.

But that’s just data collection. But then the computation, and a lot of computation at the Institute, a ton of computation at Princeton, a ton of computation at other – that’s when the confederacy turns into a bear to manage. But at least that one worked. Our papers are just hitting bioRxiv as we speak. But I’d like to declare the project was a success. It really took forever, and a ton of people. But we got a lot out of it, I think. I hope the world agrees because we did not spend two bits.

Kara: Yeah, I think they do.

Reid: Well they haven’t seen it yet, so don’t – there will be a group of order 10, probably more, papers coming out in the next year. And that’s when the world will have to say.

Kara: So, moving a little bit away from the local circuit connectomics that you’ve been talking about, rumor has it from looking at the NIH RePORTER that you’re looking at wider scales now, something like projectomics.

Reid: Yeah.

Kara: Is that a fair –

Reid: I think this is –

Kara: Can you talk about that a little bit?

Reid: Yeah. This is turning into, especially given the quip that you made earlier, it’s a nice 50-minute hour where you go into my mind to determine whether I am actually insane! Perhaps we were insane doing calcium imaging. Almost certainly we were insane jumping onto EM. Although, I think in the end it was borne out that it wasn’t foolish, even though insane.

A normal person or a normal scientist would make hay while the sun shines. We have so much wonderful data that 25, 30, 50 people are writing papers about right now. At least experimentally, I’ve moved on to the next thing. And I’m still – anyway –

Kara: Which is what?

Reid: The next thing being – let’s not get too introspective, but the next thing is there’s actually a movement, not just myself by any means, a real movement towards whole-brain anatomical connectomics, whole-brain wiring diagrams. And that takes two forms. One – both insane, but I think both are going to happen. One is – given that we and others, a small number, but others – have brought EM connectomics to the millimeter scale, to the petabyte scale, in terms of data, the whole mouse brain is only 500 times bigger. Huge technical hurdles, gigantic computational hurdles. But there are roadmaps for how to do that. And so that’s a movement that I – from the beginning, I’ve been part of a group of people pushing for it. But simultaneously, I got the crazy idea – again, not alone – Sebastian Seung has liked this idea from the beginning and there are other groups talking about it, a number of groups – that is, doing whole large brain connectomes. So the human brain is 2000 to 3000 times bigger than a mouse brain. No way in my life, no way in your life, are we going to have the whole human brain EM connectome. That’s a zettabyte of data. Anyway, it’s hard to predict the future.

But we, a group of people, believe that – go down in resolution, don’t be greedy about the size of brains reconstructed, but don’t be greedy about getting everything. So if you say you’d like to know all the long distance pathways, essentially from a cell body, say, in the cortex to its major axon that might have two, three, four, five targets throughout the brain, just trace that. And once it gets to the target area or areas, stop tracing because you won’t be able to see it anyway. Stop tracing. Don’t look at the synapses. And such a thing, rather than connectome of all the connections – in the jargon, is a “projectome” – projections from one brain area to another. And I deeply believe that’s something that we’re going to have in the coming five to ten years for experimental animal large brains. Don’t need to get into them, but I think in the next ten – at the outmost twenty, but I think the next ten years – we’ll have that for the human. And I think the reason I am obsessed is that, at some point, all of us want to work on the human because it’s important.

Kara: Absolutely.

Reid: And except for a very few counterexamples, it is true that never has a single axon, not a bunch of axons, but a single axon been traced from one place to another place in the human brain. Not one. And new techniques – whether it’s light microscopy, or synchrotron radiation, etc. – promise to be able to not trace all of them, but trace a large number of the big ones. And those number in the hundreds of millions, in the billions. And how can we not want to know that? It’s not going to be free, but I think it’s something that the world needs. And why not try?

Kara: Yeah. That sounds very exciting. I think we’re running out of time.

Reid: I do go on, don’t I?

Kara: Maybe one final question unrelated to your actual scientific enterprise, but something that I picked on that I wanted you to speak about was your writing style. All the papers you write, even though the science is so complicated, these papers are accessible to a broad audience. People from non-neuroscience disciplines can read them and say, “I kind of get what this is about.”

Even when we give journal club assignments to new grad students, they get it. And so that is something that is just under-looked, in my view: good scientific writing style. I mean, where did you get this from? We talked about management, were you good at it, and you said, well, you don’t know about that. But it’s clear, at least to me and the students here and so on, that there’s something about your writing style that – it’s just the simple elegance – can you say anything about the way you were thinking?

Reid: Thank you. Thank you for overly generous – but let me – several things. For a millisecond, I will say I’ve always – I haven’t liked writing, but I’ve always cared about writing. Write a decent amount, take enough English classes in college, all that. But mentorship, mentorship, mentorship. And yeah, maybe another deprecation, which is deeply true: Do not – anyone who’s starting out – do not let perfectionism slow down your writing as much as it’s slowed down – whatever. Get papers out.

But it’s great to be taught how to write, and I was extraordinarily lucky in my advisors scientifically, but also in terms of writing. Bob Shapley, you go back to his – or read his current papers. He writes beautifully – Bob Shapley at NYU. And he would not let a crummy sentence or a fuzzy idea get by. I wish I had the focus and whatever it is to teach that, because it’s the greatest skill that someone can impart.

And then Torsten Wiesel. I mean, no one can hold a candle to the papers that Hubel and Wiesel wrote. They were beautiful. Starting out a field, and just discursive, beautiful language.

And we were lucky when it was Jose Manuel, Judith Hirsch and I, then Marty Usrey, we were writing papers at the Rockefeller University in the early ‘90s. Torsten wasn’t even in the lab at the time. He was president of Rockefeller. But he would sit down with every draft of our – a number of papers that we wrote. He wasn’t even on them. And he would go over every sentence, every word choice. So it just takes time. Don’t be lazy about ideas.

But it’s a skill that people don’t, perhaps, pay attention to enough. But I would sadly say that people may not teach as much either. But it’s crucially important. It’s crucially important to get your ideas across to people, and it’s crucially important to keep food on the table, writing grants.

Kara: All right. I think –

Reid: I definitely have not learned how to give short answers. Torsten would do a much better job!

Kara: No, it’s all good. But I think we should end things here. And thank you for your willingness to do this. I think a lot of the readers of the journal are going to find it very helpful. And we appreciate everything you’ve done in changing the thinking of what is possible in neuroscience, crazy or not crazy. So thank you for all your efforts. And we look forward to the next projects and papers that are going to come out from your team.

Reid: Well, thank you so much, Prakash – it was really nice catching up – and for your thoughtful and overly generous ideas.

Kara: All right, thanks, Clay. Take care. Bye!

Reid: Bye!
